# Dynamics of Receptor-Mediated Nanoparticle Internalization into Endothelial Cells

**DOI:** 10.1371/journal.pone.0122097

**Published:** 2015-04-22

**Authors:** David Gonzalez-Rodriguez, Abdul I. Barakat

**Affiliations:** Laboratoire d’Hydrodynamique (LadHyX), École Polytechnique, CNRS UMR 7646, Palaiseau, France; Case Western Reserve University, UNITED STATES

## Abstract

Nanoparticles offer a promising medical tool for targeted drug delivery, for example to treat inflamed endothelial cells during the development of atherosclerosis. To inform the design of such therapeutic strategies, we develop a computational model of nanoparticle internalization into endothelial cells, where internalization is driven by receptor-ligand binding and limited by the deformation of the cell membrane and cytoplasm. We specifically consider the case of nanoparticles targeted against ICAM-1 receptors, of relevance for treating atherosclerosis. The model computes the kinetics of the internalization process, the dynamics of binding, and the distribution of stresses exerted between the nanoparticle and the cell membrane. The model predicts the existence of an optimal nanoparticle size for fastest internalization, consistent with experimental observations, as well as the role of bond characteristics, local cell mechanical properties, and external forces in the nanoparticle internalization process.

## Introduction

The pathological complications of atherosclerosis, namely heart attacks and strokes, remain the leading cause of worldwide mortality [1]. Because atherosclerosis is fundamentally a disease that involves inflammation of the endothelium, the monolayer of cells that lines the inside of blood vessels, a particularly promising idea is the use of nanoparticles as cargo vehicles for targeted delivery of anti-inflammatory agents to arterial endothelial cells. Recent studies have established that nanoparticle internalization into endothelial cells depends on a number of factors including nanoparticle size and surface functionalization [2–4]. Elucidating the basis for these observations is of primary interest.

A critical component in the development of an effective nanoparticle-based endovascular drug delivery system is the interaction between particles and the endothelial cell surface. To specifically target inflamed endothelial cells at an atherosclerotic lesion, nanoparticles can be coated with antibodies against endothelial cell adhesion molecules, such as selectins, VCAM-1, PECAM-1, or ICAM-1 [5]. Out of these different receptors, intercellular adhesion molecule-1 (ICAM-1) is a particularly relevant target, since its level of expression in vascular endothelial cells is enhanced significantly by pathological stimuli such as oxidants, cytokines, and abnormal fluid mechanical shear stresses [3]. Specifically, a 20 to 100 fold increase in ICAM-1 expression in activated over quiescent cells has been reported [6]. ICAM-1-mediated nanoparticle internalization into endothelial cells has been the subject of a number of recent experimental studies [3, 6–10]. Nanoparticles coated with anti-ICAM-1 antibodies activate a specific endocytosis pathway termed CAM-mediated endocytosis [7]. Unlike endocytosis mediated by other membrane receptors, CAM-mediated endocytosis requires multivalent binding: a single anti-ICAM-1 antibody is not internalized by an endothelial cell, whereas a particle carrying several antibodies can be internalized. CAM-mediated endocytosis is actin-dependent, but it involves different protein machineries than clathrin-mediated endocytosis, caveoli, macropynocytosis, or phagocytosis [3, 7].

In this article we develop a mathematical model to describe receptor-mediated nanoparticle internalization, specifically considering the case of ICAM-1-mediated endocytosis. Several theoretical models of nanoparticle internalization have previously been proposed. A first group of theoretical models describes receptor-mediated internalization of spherical and non-spherical particles limited by diffusion of receptors within the cell membrane [11–13]. These models assume the particle ligand density to be much larger than the cell membrane receptor density, thus making receptor diffusion towards the particle wrapping zone a limiting physical mechanism. This assumption does not appear applicable to ICAM-1-mediated nanoparticle endocytosis into inflamed endothelial cells, where receptor and ligand densities are both of the order of 1000 molecules/*μ*m^2^ [14], and diffusion of receptors thus becomes negligible. Another group of theoretical models uses energetic approaches to describe membrane wrapping of a nanoparticle (see the recent review by Bahrami et al. [15]). These models have investigated the role of nanoparticle shape and orientation [16, 17], nanoparticle deformability [18], and interactions between multiple nanoparticles [19, 20], but they often leave out the cell’s cytoplasmic rigidity and the bond formation dynamics, as well as the kinetics of the wrapping process. Recent advances in modeling the wrapping of a nanoparticle by a membrane have incorporated stochastic thermal fluctuations to study the kinetics of wrapping [21] or conformational changes of membrane proteins, which are described by particle dynamics simulations [22].

Here we develop a new theoretical model to study the kinetics of nanoparticle internalization under the following premises: (i) we consider the case where receptor and ligand density are comparable, so we neglect receptor diffusion; (ii) we include both membrane bending and viscoelastic deformation of the cytoskeleton; (iii) we account for the dynamics of bond formation under force. Unlike most of the previous theoretical models, which are based on energy formulations, we develop our model in terms of force balances. Our formulation is inspired by that proposed by Dembo et al. to study the kinetics of detachment of a membrane from a surface [23]. Our model enables us to understand how receptor-mediated internalization is affected by particle size, bond characteristics, cell mechanical properties, and external forces exerted on the nanoparticle.

## Model

**Fig 1:**
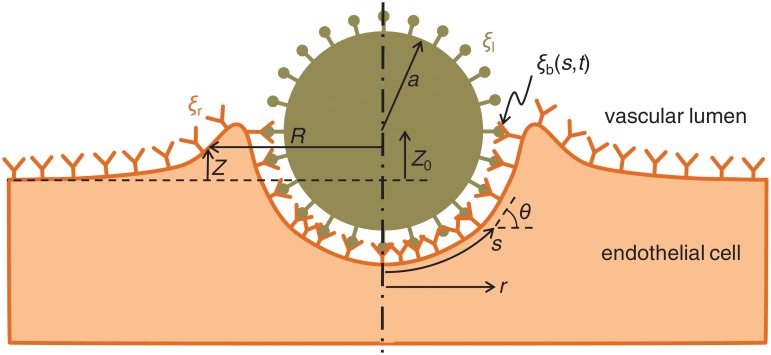
Side view of our conceptualization of the receptor-mediated internalization process. The dash-dotted line is the axis of rotational symmetry. Ligand density (*ξ*_l_) and receptor density (*ξ*_r_) are assumed comparable. Bond density (*ξ*_b_) is a function of the curvilinear coordinate, *s*, and of time, *t*. The elevation of the particle center above the initial cell membrane position is denoted by *Z*_0_(*t*). The membrane position is defined by its coordinates *R*(*s*, *t*) and *Z*(*s*, *t*).

Our conceptualization of receptor-mediated nanoparticle internalization is represented in Fig 1. We consider a three-dimensional axisymmetric geometry. Internalization is driven by the formation of bonds between ligands on the particle surface (the total density of free plus bound ligands is denoted by *ξ*_l_) and receptors on the cell membrane (the total density of free plus bound receptors is denoted by *ξ*_r_). The density of bonds is denoted by *ξ*_b_, which is a function of the curvilinear coordinate *s* and of the elapsed time *t*. The particle radius is denoted by *a*. The model aims to predict the evolution of the membrane shape, determined by the variables *R*(*s*, *t*) and *Z*(*s*, *t*); of the position of the particle, determined by the vertical elevation *Z*_0_(*t*); and of the bond density, *ξ*_b_(*s*, *t*).

### Cell membrane shape

Mechanically, the cell membrane deformation is described as the bending of an inextensible axisymmetric plate subjected to large deformations. Force equilibria in the directions tangential and normal to the membrane yield

$$\begin{array}{ccc} \frac{\partial T}{\partial s}-V\frac{\partial \theta }{\partial s}+\sigma_{t} & = & 0 \end{array}$$ (1)

$$\begin{array}{ccc} T\frac{\partial \theta }{\partial s}+\frac{\partial V}{\partial s}+\frac{T\sin\theta }{r}+\frac{V\cos\theta }{r}+\sigma_{n} & = & 0, \end{array}$$ (2)

where *T* and *V* are respectively the shear and normal forces per unit of azimuthal length of the membrane, *θ* is the angle between the membrane and the horizontal, measured in the radial plane (see Fig 1), *r* is the radial distance to the axis of symmetry, *s* is the curvilinear coordinate, and *σ*_t_ and *σ*_n_ are respectively the tangential and normal stresses acting on the membrane. In deducing these equations, we have assumed that, due to reorganization of the lipid molecules composing the membrane, the membrane tension remains isotropic, thus its value is the same in the radial and azimuthal directions.

Moment equilibrium yields

$$\begin{array}{c} V=\frac{\cos\theta }{r}(M_{s}-M_{\theta })+\frac{\partial M_{s}}{\partial s}, \end{array}$$ (3)

where *M*_s_ and *M*_θ_ are the moments in the radial and azimuthal directions. Assuming linear elastic membrane deformation, assuming that a planar cross-section of the membrane remains planar after bending, and neglecting the mean circumferential deformation of the cell membrane (i.e., neglecting stresses due to membrane stretching), we deduce the following relations between the moments and the membrane shape:

$$\begin{array}{ccc} M_{s} & = & -B\;(\frac{\partial \theta }{\partial s}+\nu \frac{\sin\theta }{r}) \end{array}$$ (4)

$$\begin{array}{ccc} M_{\theta } & = & -B\;(\frac{\sin\theta }{r}+\nu \frac{\partial \theta }{\partial s}), \end{array}$$ (5)

where *B* = *E*_m_ *h* ^3^/(12(1 − *ν* ^2^)) is the membrane bending modulus, *h* is the membrane thickness, *E*_m_ is the Young’s modulus of the membrane, and *ν* its Poisson’s ratio. Using Eqs 3–5, the force equilibria given by Eqs 1 and 2 can be rewritten in terms of two unknowns, *θ* and *T*. For reasons of numerical stability, we project the force equilibrium equations onto the radial and vertical axes. The projected equations are:

$$\begin{array}{ccc} & & \cos\theta \frac{\partial T}{\partial s}-T\sin\theta \frac{\partial \theta }{\partial s}-\frac{T\sin^{2}\theta }{r}+B\;[\sin\theta \frac{\partial^{3}\theta }{\partial s^{3}}\\ & & \;+\frac{2\sin\theta \cos\theta }{r}\frac{\partial^{2}\theta }{\partial s^{2}}+\cos\theta \frac{\partial \theta }{\partial s}\frac{\partial^{2}\theta }{\partial s^{2}}\\ & & \;+\frac{\cos(2\theta )}{r}{\left(\frac{\partial \theta }{\partial s}\right)}^{2}-\frac{\sin\theta (\sin^{2}\theta -2\cos^{2}\theta )}{r^{2}}\frac{\partial \theta }{\partial s}\\ & & \;+\frac{\sin^{2}\theta \cos^{2}\theta }{r^{3}}]+\sigma_{r}=0 \end{array}$$ (6)

and

$$\begin{array}{ccc} & & \sin\theta \frac{\partial T}{\partial s}+T\cos\theta \frac{\partial \theta }{\partial s}+\frac{T\sin\theta \cos\theta }{r}+B\;[-\cos\theta \frac{\partial^{3}\theta }{\partial s^{3}}\\ & & \;-\frac{2\cos^{2}\theta }{r}\frac{\partial^{2}\theta }{\partial s^{2}}+\sin\theta \frac{\partial \theta }{\partial s}\frac{\partial^{2}\theta }{\partial s^{2}}\\ & & \;+\frac{\sin(2\theta )}{r}{\left(\frac{\partial \theta }{\partial s}\right)}^{2}+\frac{\cos\theta (\cos^{2}\theta -2\sin^{2}\theta )}{r^{2}}\frac{\partial \theta }{\partial s}\\ & & \;-\frac{\sin\theta \cos^{3}\theta }{r^{3}}]+\sigma_{v}=0, \end{array}$$ (7)

where *σ*_r_ and *σ*_v_ are respectively the radial and vertical stresses acting on the membrane.

### Stresses exerted on the membrane

**Fig 2:**
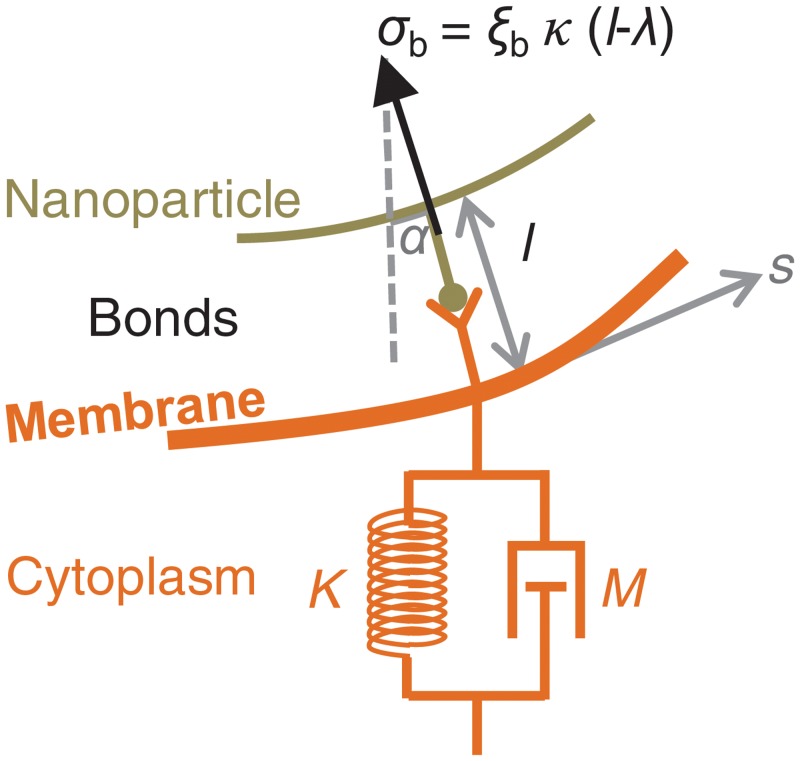
Schematic of the stresses exerted on the membrane. The membrane’s top face sustains a stress *σ*_b_ arising from bond deformation. The bonds behave as linear elastic springs of constant *κ* and undeformed length *λ*. In addition, if the membrane and the particle get very close, a short-range repulsion force (not represented in the figure) prevents particle-membrane interpenetration. The membrane’s bottom face sustains a stress arising from the viscoelastic deformation of the cell’s cytoplasm, which is modeled as a Kelvin-Voigt material of elastic constant *K* = *E*/*a* and viscous constant *M* = *μ*/*a*.

Stresses exerted on the membrane arise from interactions with the nanoparticle and with the cell’s cytoplasm, as represented in Fig 2. We consider three types of stresses acting on the cell membrane. The first type are the stresses due to viscoelastic deformation of the cytoplasm. The cytoplasm is treated as a Kelvin-Voigt material, i.e., a system comprising an elastic spring, of constant *K* = *E*/*a*, in parallel with a damper, of constant *M* = *μ*/*a*. Here, *E* and *μ* are respectively the Young’s modulus and the viscosity of the cytoplasm, and *a* is the particle radius. The cytoplasm is assumed to exert a purely vertical stress on the membrane. The model does not account for the active forces generated by cytoskeletal dynamics, such as those due to actin polymerization, which develop over a time scale of several minutes [3]. Thus, strictly speaking, the model describes only the early stages of internalization, up to the first few minutes. The second type of stresses are due to the deformation of the bonds between the membrane and the particle. The bonds are treated as linear elastic springs of constant *κ* and undeformed length *λ*. Bond orientation is assumed to point towards the nanoparticle center. We denote by *α* the angle between the bond direction and the vertical (see Fig 2). The total stress exerted locally by the bonds is proportional to the local bond density, *ξ*_b_. The bonds can exert larger traction forces than compression forces, since the latter are limited by the bond length remaining positive, *l* ≥ 0. Therefore, to avoid membrane-particle interpenetration, we need a third type of stresses that can provide sufficiently large contact forces between the particle and the membrane. We thus introduce a short-range repulsion force using the formulation proposed by [24]. Adding these three components, the total radial and vertical stresses on the membrane are:

$$\begin{array}{ccc} \sigma_{r} & = & -\xi_{b}\kappa \left(l-\lambda \right)\sin\alpha \\ & & +\frac{\gamma }{l}(\frac{1}{l}+\frac{1}{\tau })\exp\{-\frac{l}{\tau }\}\sin\alpha \end{array}$$ (8)

$$\begin{array}{ccc} \sigma_{v} & = & (-KZ-M\frac{\partial Z}{\partial t})\cos\theta +\xi_{b}\kappa \left(l-\lambda \right)\cos\alpha \\ & & -\frac{\gamma }{l}(\frac{1}{l}+\frac{1}{\tau })\exp\{-\frac{l}{\tau }\}\cos\alpha , \end{array}$$ (9)

where *Z* is the membrane elevation with respect to the undeformed position, *t* is the time, *l* is the distance between the membrane and the particle, measured in the direction pointing towards the particle center, and *γ* < 1 nN and *τ* ≈ 5 − 30 nm are constants in the short-range repulsion force formulation [24].

### Membrane-particle bonds

The evolution of the bond density at a given membrane position, *s*, and time, *t*, is computed using Bell’s model:

$$\begin{array}{c} \frac{\partial \xi_{b}}{\partial t}=K_{f}\;(\xi_{l}-\xi_{b})(\xi_{r}-\xi_{b})-K_{d}\xi_{b}, \end{array}$$ (10)

where *ξ*_*b*_(*s*, *t*) is the instantaneous, local bond density, *ξ*_*l*_ and *ξ*_*r*_ are respectively the total ligand and receptor densities, and *K*_*f*_ and *K*_*d*_ are respectively the bond formation and dissociation coefficients. Using the formalism proposed by [25], we assume *K*_*f*_ to be constant and *K*_*d*_ to depend on the force applied to the bonds through:

$$\begin{array}{c} K_{d}=K_{d}^{\left(0\right)}\exp\{\frac{\kappa |l-\lambda |x_{b}}{k_{B}𝓣}\}, \end{array}$$ (11)

where $K_{\text{d}}^{\left(0\right)}$ is the baseline value at zero force, *k*_B_ ≈ 1.38 ⋅ 10^−23^ J/K is the Boltzmann constant, 𝓣≈310K is the temperature, *κ* is the bond spring constant, and *x*_b_ is a constant length that characterizes the height of the energy barrier needed to break a bond [25, 26]. Eq 11 assumes the dissociation constant to depend on forces applied to the bonds irrespective of their sign (tension or compression). The rationale underlying this assumption is that there is an optimal distance *λ* for ligand-receptor interaction, whereas when receptors and ligands are compressed into closer distances to each other, they may be forced into conformations that are suboptimal for the interaction between their respective binding sites. Thus, we expect the dissociation constant to increase with an applied compressive force, as is the case under a tensile force.

### Particle position

The vertical particle position with respect to the membrane, *Z*_0_, is determined by balancing the forces acting on the particle: the bond forces, the membrane-particle repulsive forces, and an external vertical force that may be exerted directly on the particle, *F*_v_. Vertical force equilibrium yields:

$$\begin{array}{ccc} & & F_{v}-\int_{0}^{\infty }[\xi_{b}\kappa \left(l-\lambda \right)\cos\alpha \\ & & \;-\frac{\gamma }{l}(\frac{1}{l}+\frac{1}{\tau })\exp\{-\frac{l}{\tau }\}\sin\alpha ]\;2\pi rdr=0. \end{array}$$ (12)

The equation is implicit in *Z*_0_, since *l* and *α* depend on *Z*_0_ through straightforward geometric relations.

### Model parameters and nondimensionalization

The model parameters and their typical values are summarized in Table 1. The bond characteristics correspond to values found in the literature for ICAM-1 receptor-antibody pairs. The tabulated binding constants, *K*_f_ and $K_{\text{d}}^{0}$, are typical values for membrane-bound ICAM-1 receptors and surface-adsorbed anti-ICAM-1 antibody ligands. These values are estimated from the experimental results by [27], which, it should be noted, were performed using a soluble form of ICAM-1 and surface-adsorbed anti-ICAM-1 antibodies. To deduce the tabulated value range for *K*_f_ and $K_{\text{d}}^{0}$, we apply the relationship between soluble and surface binding kinetics proposed by [25]. To apply Bell’s formulas, we assume a diffusion coefficient for soluble ICAM-1 of *D*_s_ = 50 *μ*m^2^/s [25], a diffusion coefficient for membrane-bound ICAM-1 in the range *D*_*m*_ = 0.01 − 0.4 *μ*m^2^/s [28, 29], and a maximum distance for receptor-ligand interaction of 1 nm [25]. The value of the repulsion constant *γ* is chosen so that repulsion becomes negligible (compared to bond elasticity) at a distance equal to the bond’s equilibrium length, *λ*. This leads us to set *γ* = 1 fN. We note that our model results are insensitive to changes in *γ* of up to one order of magnitude.

**Table 1:** Typical values of the model parameters.

parameter	symbol	best estimate	range	reference
particle radius	*a*	0.1 *μ*m	0.05 − 2	[3]
ligand density	*ξ*_l_	10^3^ *μ*m^−2^	300 − 7000	[14]
receptor density	*ξ*_r_	10^3^ *μ*m^−2^	100 − 5000	[8, 14]
bond elasticity	*κ*	10^−2^ N/m	10^−5^ − 1	[14, 24, 38, 39]
bond length	*λ*	20 nm	15 − 35	[14, 38, 40]
repulsion constant	*γ*	1 fN	0 − 10^6^	[24]
repulsion length	*τ*	5 nm	5 − 20	[24]
binding rate	*K*_f_	10^−2^ *μ*m^2^/s	0.002 − 0.06	[27]
bond equilibrium constant	$K_{\text{d}}^{\left(0\right)}/K_{\text{f}}$	0.05 *μ*m^−2^	0.02 − 0.10	[27]
Bell’s length constant	*x*_b_	0.5 nm	0.1 − 1.0	[25, 41]
membrane tension	*T*_0_	30 pN/*μ*m	10 − 50	[42–44]
cytoplasm elasticity	*E*	1 kPa	1 − 7	[45, 46]
cytoplasm viscosity	*μ*	1 kPa ⋅ s	0.5 − 6	[47–49]
membrane bending	*B*	10^−19^ J	10^−20^ − 10^−19^	[11, 23]

The equations are nondimensionalized using the particle radius, *a* ≈ 100 nm, as the unit of length and the ligand density, *ξ*_l_ ≈ 1000 molecules/*μ*m^2^, as the unit of bond density. Since internalization is driven by bond formation, the unit of stress is taken as $\xi_{\text{l}}k_{\text{B}}𝓣/x_{\text{b}}\approx 10^{4}\;\text{Pa}$, which is the bond stress corresponding to a characteristic bond elongation of $k_{\text{B}}𝓣/(\kappa x_{\text{b}})$. There are two time scales, given by the rate of bond formation and by the rate of viscoelastic cytoplasmic deformation. Since the cytoplasm’s viscoelastic time scale, *μ*/*E* ≈ 1 s, is usually longer than the bond formation time scale, 1/(*K*_f_ *ξ*_l_) ≈ 0.1 s, the former is chosen as the unit of time for nondimensionalization. By nondimensionalizing the equations with the aforementioned units of length, bond density, stress, and time, we deduce the nondimensional parameters characterizing the problem, which are listed in Table 2. The tabulated values show that the three forces resisting deformation, due to cell viscoelasticity (*C*_E_), membrane bending (*C*_B_), and membrane tension (${\hat{T}}_{0}$), are of comparable magnitude, and therefore they should all be accounted for in the model. The nondimensional parameter *t*_b_ is the ratio of the bond formation time to the viscoelastic time, which is typically smaller than 1, since bond formation is fast compared to viscoelastic deformation.

**Table 2:** Nondimensional coefficients and typical values.

parameter	definition	typical value
${\hat{\xi }}_{\text{r}}$	*ξ*_r_/*ξ*_l_	1
${\hat{K}}_{\text{r}}$	$K_{\text{d}}^{\left(0\right)}/(K_{\text{f}}^{\left(0\right)}\xi_{\text{l}})$	5 ⋅ 10^−5^
$\hat{\lambda }$	*λ*/*a*	0.2
$\hat{\tau }$	*τ*/*a*	0.05
*C*_E_	*Ex*_b_/(*ξ*_l_ *κk*_B_𝓣)	10
*C*_B_	*Bx*_b_/(*ξ*_l_ *a* ^3^ *κk*_B_𝓣)	1
*C*_*ξ*_	*aκx*_b_/(*k*_B_𝓣)	100
*C*_rep_	*γx*_b_/(*ξ*_l_ *a* ^2^ *κk*_B_𝓣)	10^−3^
${\hat{T}}_{0}$	*T*_0_ *x*_b_/(*ξ*_l_ *aκk*_B_𝓣)	3
*C*_K_	*κax*_b_/(*k*_B_𝓣)	100
*t*_b_	*E*/(*μK*_f_ *ξ*_l_)	0.1

### Numerical solution

We need to solve the system formed by Eqs 6 and 7, which determine the instantaneous membrane shape; Eq 12, which determines the instantaneous particle position; and Eq 10, which determines the evolution of the bond density. The equations are solved iteratively in time. Initially (*t* = 0), the membrane is assumed flat (*R*(*s*, 0) = 0, *Z*(*s*, 0) = 0), the particle is located above the membrane at *Z*_0_(*t* = 0) = *a* + *λ*, and the particle is assumed to be weakly attached to the membrane through an initial bond density *ξ*_b_(*s*, 0) = 0.01*ξ*_l_(1 − 5*s*/*a*) for *s* < 0.2*a*, and *ξ*_b_(*s*, 0) = 0 otherwise. Provided that the initial bond distribution is weak, and due to swift bond formation at early times, the precise structure of the initial bond distribution has virtually no effect on the model results presented here. At each time step, *t* + Δ*t*, we start by updating the bond density. With the membrane shape and particle position from the previous time, *t*, we analytically solve Eq 10 to compute the updated bond density, *ξ*_b_(*s*, *t* + Δ*t*). The time increment Δ*t* is variable, and it is chosen by the solver so that the change in bond density is smaller than given relative (2%) and absolute (0.02*ξ*_l_) thresholds. The values of these thresholds have been chosen so that further reductions of the thresholds do not alter the final results by more than 5%. The time steps obtained in this manner are very small at early times (of the order of 10^−6^ time units), when bond formation progresses rapidly, and significantly larger at late times (of the order of 0.05 time units), when the internalization process approaches the final equilibrium state. Once the updated bond density at *t* + Δ*t* has been obtained, the new membrane shape and particle position are calculated by solving Eqs 6, 7, and 12. To obtain the membrane shape, axial symmetry conditions are imposed at the membrane center (*s* → 0): *R* = 0, *θ* = 0, and ∂^2^ *θ*/∂*s* ^2^ = 0. At the outer edge of the membrane, *s* = *s*_max_, the following boundary conditions are imposed: *Z* = 0, *M*_s_ = 0, *T* cos*θ* − *V* sin*θ* = *T*_0_. We note that the radial position of the outer edge, *R*(*s*_max_), is left free; it will decrease during the wrapping process to satisfy the membrane inextensibility assumption. The outer edge is taken as *s*_max_ = 20 *a*. We have verified that further increasing the value of *s*_max_ does not affect the simulation results. Moreover, we also verify that the membrane has recovered its planar horizontal shape at *s* = *s*_max_, i.e., that *dZ*/*ds* ≈ 0. Eqs 6 and 7 are linearized using a Newton-Raphson iteration scheme, then discretized in space and time using a finite difference scheme. The time step is taken equal to Δ*t* defined above. The spatial grid is not uniform. Rather, the grid size is given by Δ*s* = 0.04 *a*/*g*(*s*), with *g*(*s*) = 1 + 10 exp{−*s* ^2^/(2*a* ^2^)}. This relation yields a grid size that is about 10 times finer under the particle center (Δ*s*(*s* → 0) ≈ 4 ⋅ 10^−3^ *a*) than far from the particle (Δ*s*(*s*_max_) ≈ 4 ⋅ 10^−2^ *a*). We have verified that further grid refinement does not change the final results by more than 5%. The results are advanced in time following this scheme until the membrane shape converges to a final equilibrium wrapping state, which is typically reached in less than 40 time units. The solution algorithm is implemented in Matlab R2012a.

## Results

**Fig 3:**
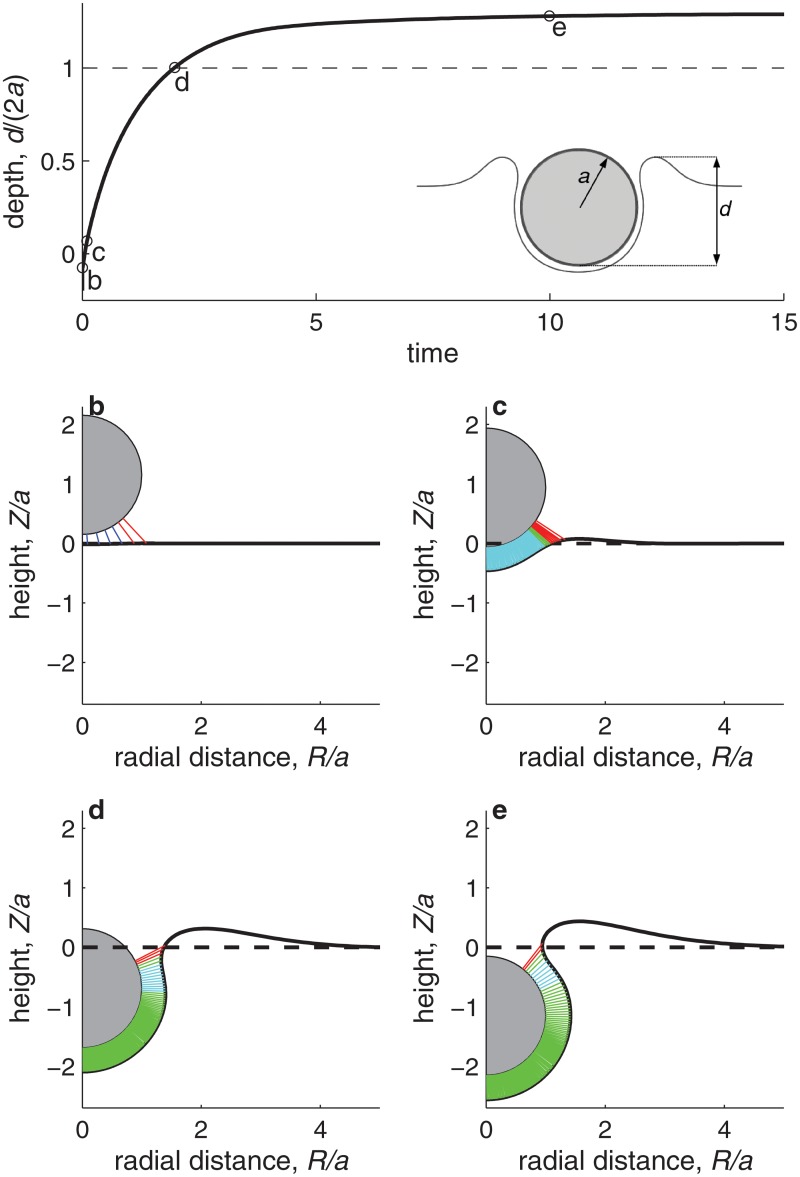
Kinetics of nanoparticle internalization. **a.** Evolution of the particle internalization, measured by the depth-to-diameter ratio, *d*/(2*a*) (see inset), as a function of the elapsed non-dimensional time (one nondimensional time unit corresponds typically to one second). The computation is performed with *a* = 50 nm, $K_{\text{d}}^{\left(0\right)}/K_{\text{f}}=0.01\;\mu {\text{m}}^{-2}$ and otherwise with the best estimate of the parameter values listed in Table 1. The horizontal dashed line corresponds to the value *d*/(2*a*) = 1, which is the threshold where we define a particle as internalized. Four snapshots of the internalization process, indicated on plot **a** by circle markers denoted by ‘b’ to ‘e’ and corresponding to times *t* = 5 ⋅ 10^−3^, *t* = 10^−2^, *t* = 2.0, and *t* = 10.0 s, respectively, are presented in the plots **b** to **e**. Each snapshot shows the instantaneous membrane shape (full line), the initial membrane shape (dashed line), the particle (gray shaded circle), and the bonds formed between the particle and the membrane (short color lines). The density of color lines is proportional to the bond density. Dark blue, cyan, green, and red lines correspond respectively to bonds that are strongly compressed (*l* < 0.9*λ*), slightly compressed (0.9*λ* ≤ *l* < *λ*), slightly stretched (*λ* < *l* ≤ 1.1*λ*), or strongly stretched (*l* > 1.1*λ*).

Fig 3 illustrates the typical internalization kinetics predicted by the model. Fig 3a shows how the internalization process, quantified through the ratio of the particle’s penetration depth *d* to the particle diameter 2*a* (see figure inset), evolves in time. The process first advances very rapidly, and then it slows down to asymptotically reach a maximum particle penetration, at which an equilibrium is reached and internalization stops. At this final state, the energy gained in bond formation by further wrapping of the particle does not compensate the energetic cost to further bend the membrane and deform the cytoplasm. The final *d*/(2*a*) value depends thus on the local cell deformability, as we will discuss below. Fig 3b–3e show snapshots of the internalization process. We can distinguish three phases. The early phase, up to snapshot 3c, is a short phase characterized by rapid bond formation and development of firm adhesion between the particle and the cell membrane. In this early phase, the bond density is not yet sufficiently high to fully bend the membrane around the particle, and thus we observe bonds that are both under strong compression (dark blue) and under strong tension (red; see snapshot 3b). The second phase, between snapshots 3c and 3d, is characterized by a relatively constant density of bonds along the particle-membrane contact region. These bonds, represented in cyan and green, are all close to their neutral state (i.e., their length *l* is close to the equilibrium value *λ*). Over this contact region, the membrane is wrapped around the particle and follows its spherical shape. Internalization progresses driven by bond formation within a small leading region, where the bonds are under strong tension (bonds represented in red). The final phase, represented by the snapshot 3e, corresponds to internalization approaching the final equilibrium state, where further wrapping is no longer favorable.

**Fig 4:**
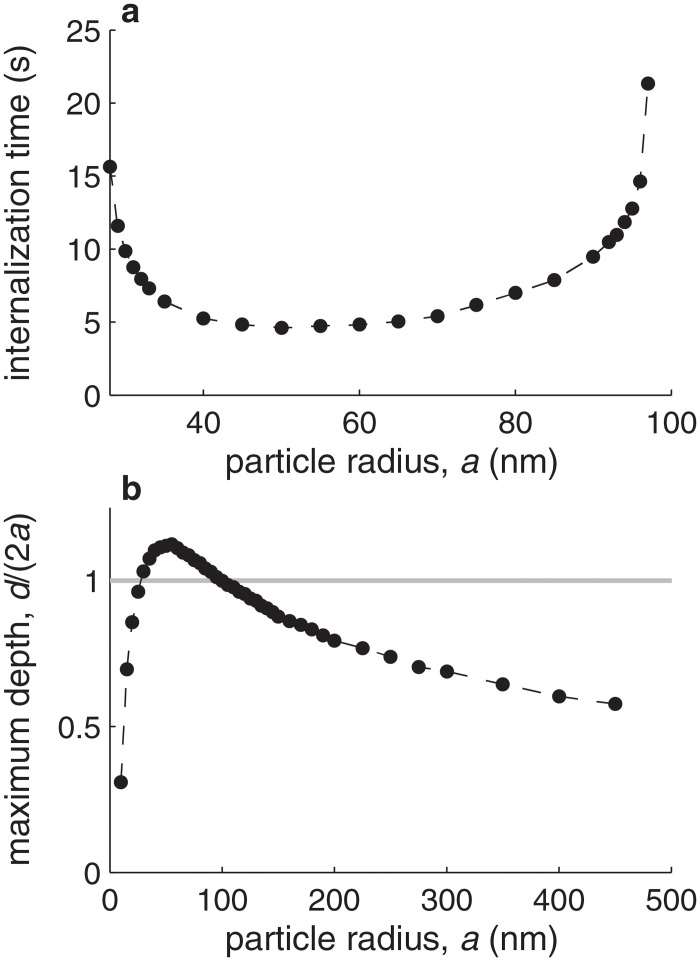
Effect of particle size. **a.** Time required for particle internalization (defined as the time required to reach *d*/(2*a*) = 1) as a function of the particle radius, *a*, for all parameter values (other than *a*) equal to their best estimate (Table 1). The circles indicate the cases for which computations have been performed, and the dashed line joins the circles to guide the eye. Below *a* = 30 nm and above *a* = 110 nm, internalization (*d*/(2*a*) = 1) is not reached. **b.** Maximum internalization depth, *d*/(2*a*), which the internalization process converges to at sufficiently long time. The gray horizontal line corresponds to *d*/(2*a*) = 1, which we define as the threshold above which the particle is considered to be internalized.

Fig 4 illustrates the effect of the nanoparticle size on the internalization process. Fig 4a shows how the time required for internalization depends on the nanoparticle radius. We define the time for internalization as the time required for reaching *d*/(2*a*) = 1. For typical parameter values (the *best estimates* in Table 1), our model predicts an optimal radius at which internalization is fastest of *a* ≈ 50 nm, which is consistent with experimental observations [4]. The time required for internalization remains comparable for a range of radii between 30 and 90 nm. Outside this range, the time required to reach *d*/(2*a*) = 1 becomes asymptotically longer, and at *a* = 20 nm or *a* = 100 nm the stage *d*/(2*a*) = 1 is no longer reached, indicating that internalization efficiency decreases significantly for very small and very large particles. Fig 4b shows the maximum value of *d*/(2*a*) that is reached as a function of the particle size. When the particle radius decreases below *a* = 20 nm, *d*/(2*a*) rapidly approaches zero, indicating that no particle wrapping occurs. This suggests that receptor-mediated internalization becomes impossible for small radii. In contrast, the maximum amount of wrapping attained for particles larger than *a* = 100 nm decreases slowly with increasing particle size, indicating a larger degree of membrane wrapping around the particle and thus suggesting that receptor-mediated internalization may still be possible up to a micrometric particle size (i.e., *a* ≈ 500 nm), as observed in experiments [3].

**Fig 5:**
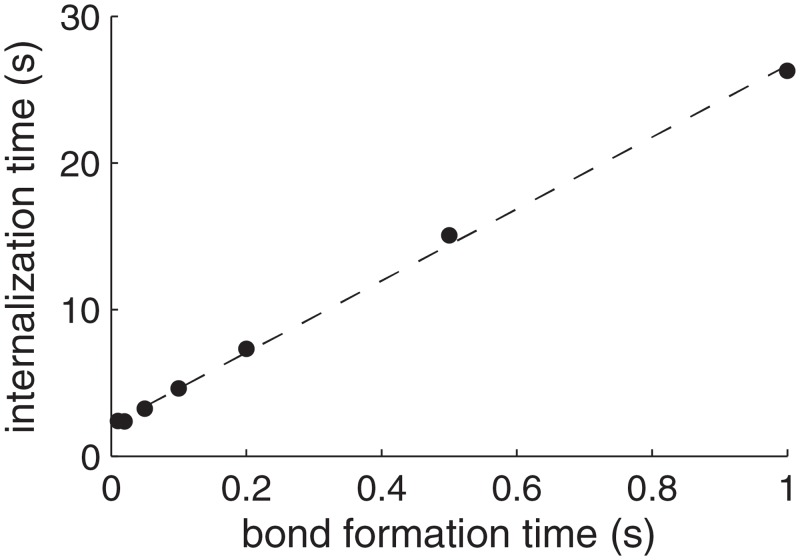
Particle internalization time as a function of the bond formation time, *t*_b_. Both times are normalized by the viscoelastic time *μ*/*E*, and can be interpreted as given in seconds if *μ*/*E* = 1 s. The circles correspond to the computations, performed with parameter values (other than *a* = 50 nm and than *K*_f_, which is variable since it determines *t*_b_) equal to their best estimate (Table 1). The straight dashed line is a fit through the data points.

Fig 5 shows the dependence of the particle internalization time (defined as the time to reach *d*/(2*a*) = 1) on the time required for bond formation, *t*_b_. In the figure both axes are expressed in seconds, but they can equivalently be regarded as normalized by the viscoelastic time scale, *μ*/*E*, which we have adopted as our time unit, and whose typical value is indeed of the order of 1 s. The figure shows that the time required for particle internalization increases proportionally to both the bond formation time and to the viscoelastic time. Interestingly, whereas both the viscoelastic time and the bond formation time are at most of the order of a second, the particle internalization time is much longer, of the order of a few tens of seconds and up to minutes, as observed in experiments [3]. The reason for the emergence of this longer internalization time scale is that, whereas bond formation induces deformations at the scale of the bond length, internalization requires deformations at the length scale of the particle. Thus, the ratio of time scales of particle internalization to bond formation is proportional to the ratio of length scales of the particle to the bond.

**Fig 6:**
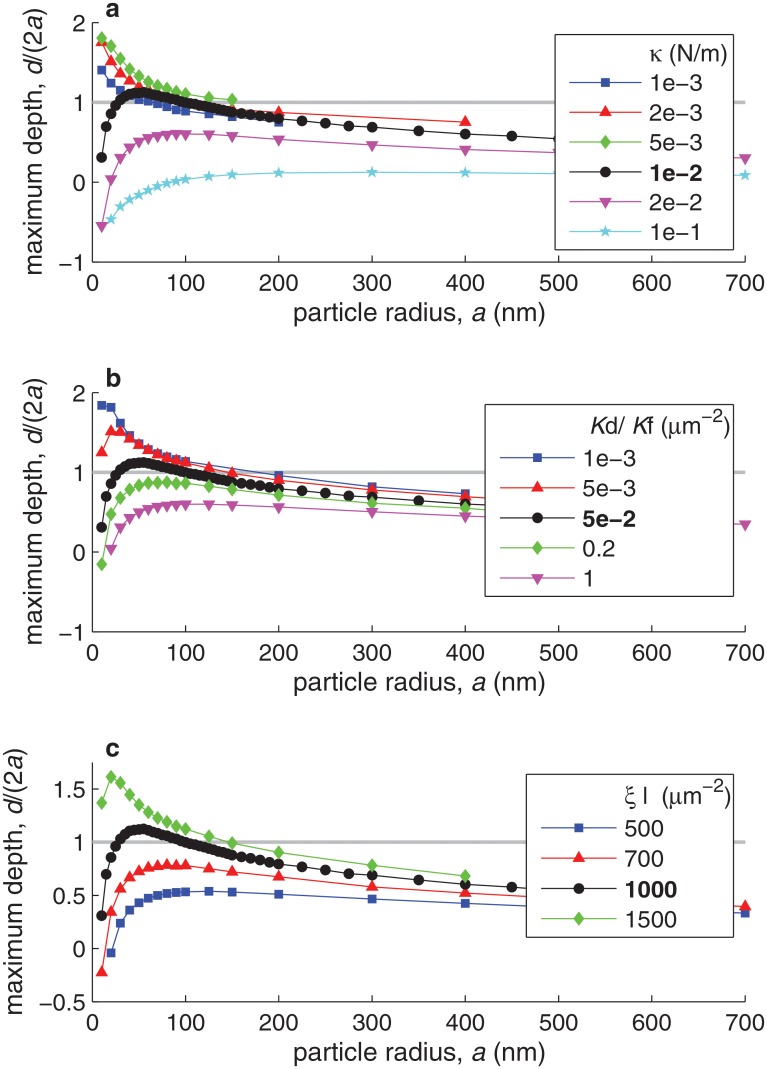
Effect of bond properties on the maximum internalization depth as a function of the particle radius, *a*. Different marker shapes and colors correspond to different values of the bond spring constant *κ* (in plot **a**), of the bond reaction constant $K_{\text{d}}^{\left(0\right)}/K_{\text{f}}$ (in plot **b**), or of the ligand density *ξ*_l_ (in plot **c**). In all plots, the black curve corresponds to the *best estimate* of the varied parameter (*κ* = 10^−2^ N/m, $K_{\text{d}}^{\left(0\right)}/K_{\text{f}}=5\cdot 10^{-2}\;\mu {\text{m}}^{-2}$, and *ξ*_l_ = 10^3^ *μ*m^−2^, respectively). In each plot, all parameter values not indicated are taken equal to their best estimates (Table 1), except for *ξ*_r_ = *ξ*_l_ in plot **c**. The gray horizontal line indicates the internalization threshold, *d*/(2*a*) = 1.

Fig 6 shows curves of maximum particle internalization depth (maximum *d*/(2*a*)) versus particle radius (*a*) for different values of several bond parameters. Fig 6a shows the effect of changes in the bond spring constant, *κ*. Interestingly, there appears to be an optimal value of *κ*, of about 5 ⋅ 10^−3^ N/m, for which particle internalization is most efficient. The existence of this optimum arises from two opposing effects of bond rigidity, *κ*, on particle wrapping. On the one hand, reducing bond rigidity reduces the force provided by each bond to deform the cell and wrap the nanoparticle. On the other hand, increasing bond rigidity makes the bond dissociation constant increase very rapidly with bond length (due to Eq 11), thus impairing bond formation. Fig 6b and 6c respectively show the effect of changes in the bond reaction constant, $K_{\text{d}}^{\left(0\right)}/K_{\text{f}}$, and in the ligand density, *ξ*_l_, which is in all cases assumed equal to the receptor density, *ξ*_r_. As expected, particle internalization becomes more efficient with decreasing $K_{\text{d}}^{\left(0\right)}/K_{\text{f}}$ and with increasing *ξ*_l_, since in both cases the available energy gain from bond formation increases.

**Fig 7:**
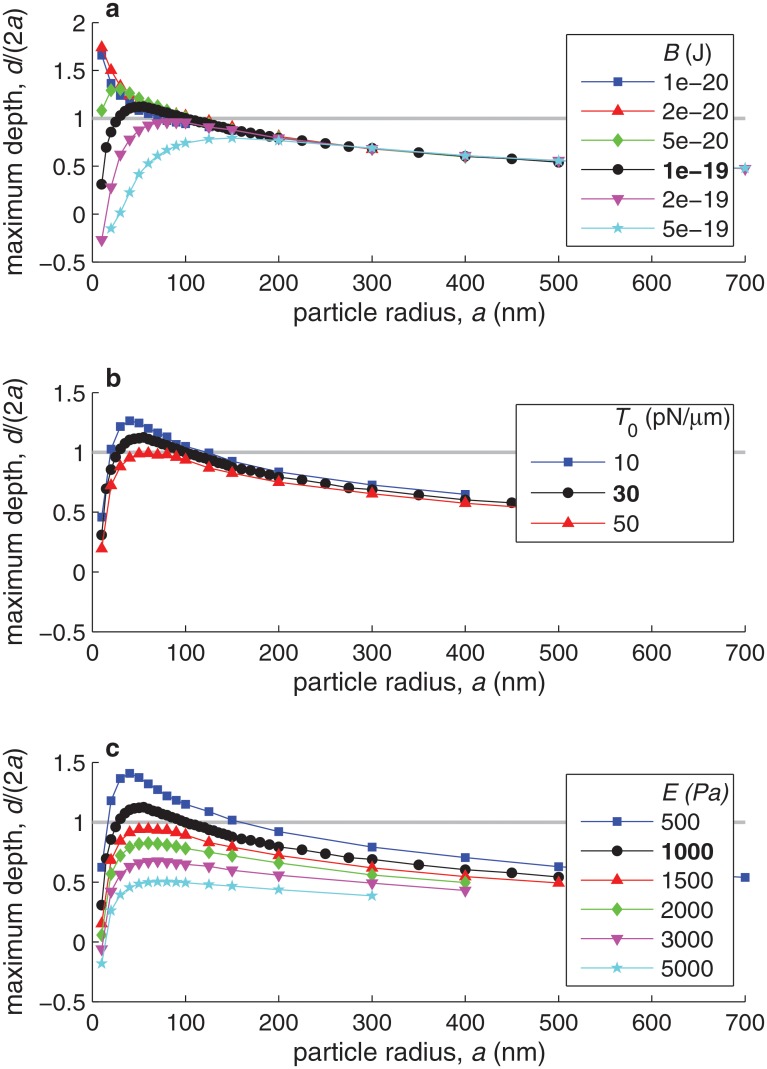
Effect of cell mechanical properties on the maximum internalization depth as a function of the particle radius, *a*. Different marker shapes and colors correspond to different values of the membrane bending modulus *B* (in plot **a**), of the initial membrane tension *T*_0_ (in plot **b**), or of the cell’s Young’s modulus *E* (in plot **c**). In each plot, the black curve corresponds to the *best estimate* of the varied parameter (*B* = 10^−19^ J, *T*_0_ = 30 pN/*μ*m, and *E* = 10^3^ Pa, respectively). On each plot, all parameter values not indicated are taken equal to their best estimates (Table 1). The gray horizontal line indicates the internalization threshold, *d*/(2*a*) = 1.

Fig 7 shows curves of maximum particle internalization depth (maximum *d*/(2*a*)) versus particle radius (*a*) for different values of the cell membrane properties (its bending rigidity *B* and its tension *T*_0_) and of the cytoplasm properties (its Young’s modulus *E*). As expected, internalization is favored by a smaller resistance to cell deformation, thus by smaller *B*, *T*_0_, or *E*. However, changes in these different parameters affect internalization differently. Within the range of physiological values, the effect of changes in *T*_0_ on internalization are much smaller than those of changes in *B* or in *E*. Changes in *B* strongly affect the internalization of smaller particles, since membrane bending stresses, which scale as *B*/*a* ^3^, are the dominant resistance to small particle internalization. Large particle internalization, on the other hand, is virtually independent of the value of *B* and strongly controlled by the value of *E*. Fig 7 thus shows that cytoplasmic rigidity becomes the dominant resisting mechanism at large *a*, as one would expect from comparing the dependence on *a* of the nondimensional coefficients *C*_E_, *C*_B_, and ${\hat{T}}_{0}$ in Table 2.

**Fig 8:**
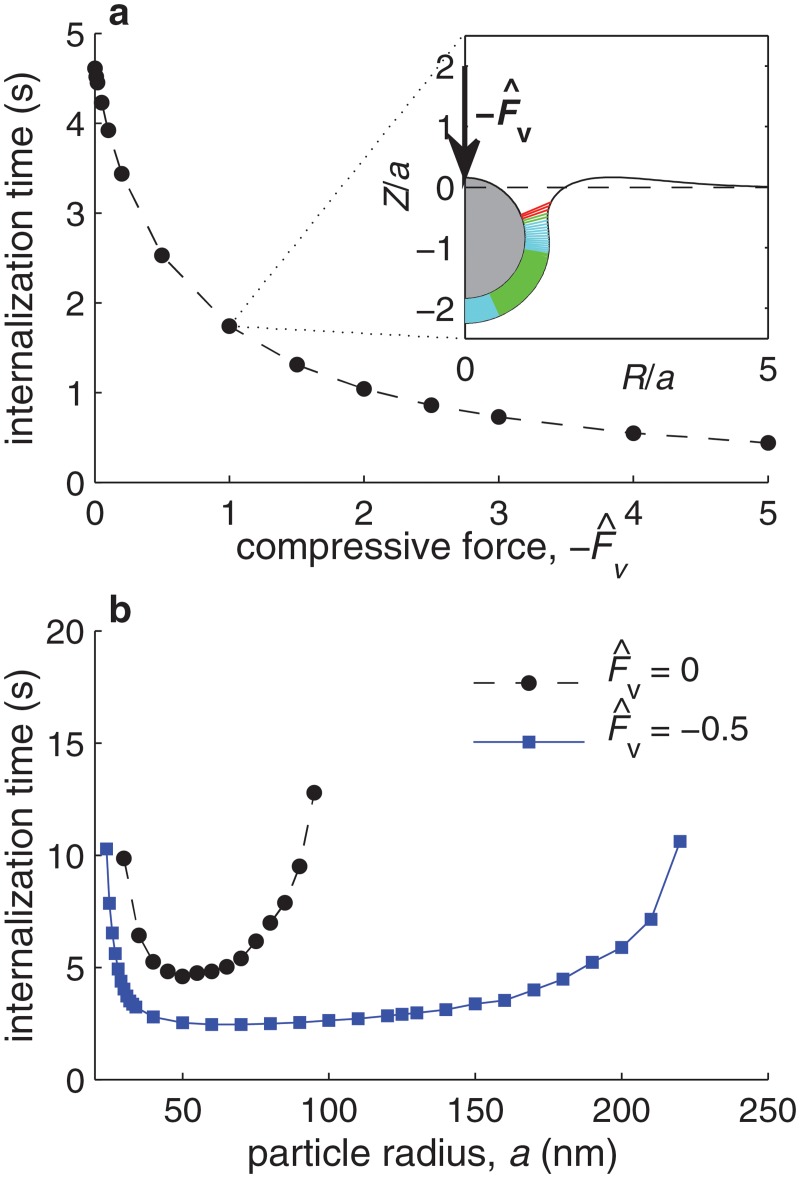
Effect of a vertical force on internalization. **a.** Time required for particle internalization (defined as the time required to reach *d*/(2*a*) = 1) as a function of a normalized vertical compressive force, 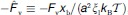, for *a* = 50 nm and all other parameter values equal to their best estimates (Table 1). The inset shows a snapshot of the membrane deformation and particle position for the case ${\hat{F}}_{\text{v}}=-1$. The snapshot corresponds to the time when *d*/*a* = 1. **b.** Time required for particle internalization (defined as the time required to reach *d*/(2*a*) = 1) as a function of the particle radius *a* in the absence of force (black circles) and in the presence of a normalized force ${\hat{F}}_{\text{v}}=-0.5$ (blue squares).

Fig 8a shows the dependence of the time required for the internalization of a particle of radius *a* = 50 nm on the magnitude of a normal, nondimensional force, ${\hat{F}}_{\text{v}}\equiv F_{\text{v}}x_{\text{b}}/(a^{2}\xi_{\text{l}}k_{\text{B}}𝓣)$, exerted onto the particle (see figure inset). Consistent with experimental observations using magnetic nanoparticles subjected to a magnetic field [30], we predict the internalization time to decrease with the applied force. The figure shows that the nondimensional force required to significantly decrease the time for particle internalization is of the order of one nondimensional unit, which for typical parameter values corresponds in dimensional terms to a stress applied onto the particle of about 2 kPa (or equivalently a total force of about 20 pN exerted onto the 50 nm particle). Fig 8b illustrates the effect on the internalization time of applying a nondimensional force ${\hat{F}}_{\text{v}}=-0.5$ onto particles of different radii *a*. Dimensionally, ${\hat{F}}_{\text{v}}=-0.5$ corresponds to a stress of about 1 kPa applied onto the particle top surface. The figure shows that a compressive stress more significantly improves the internalization of larger particles. As discussed above, the internalization of small particles is mainly limited by the membrane bending rigidity, whereas the internalization of large particles is mainly limited by the rigidity of the cell’s cytoplasm. Because the main effect of a compressive force is to deform the cell’s cytoplasm, external compressive stresses particularly facilitate the internalization of large particles. The figure thus suggests that external forces acting on nanoparticles—induced for example by electric charges, magnetic fields, or the flow pressure—can favor the internalization of larger particles.

We have also investigated the effect of a shear flow on particle internalization. To this end, we have developed a two-dimensional version of the model, where the particle shape becomes an infinitely long cylinder. The two-dimensional model allows us to consider the fore-aft asymmetric case where a horizontal force and torque act on the nanoparticle. We found that the flow-induced drag only affects the early stage of particle adhesion to the membrane, when the particle is most exposed to the flow. A sufficiently strong flow drag will rapidly detach the particle and no internalization will take place. However, if the initial adhesion is sufficiently strong to withstand the flow drag, the flow does not affect the particle internalization dynamics, which are then virtually identical to the no-flow case. The effect of the flow on the results is thus bimodal and abrupt, and it depends critically on the initial bond distribution. This result is consistent with numerous studies that have described the important role of flow in particle adhesion [14, 31–33]. However, recent studies have shown that flow also has a direct effect on nanoparticle internalization [9, 34]. Our two-dimensional computations of internalization under shear flow suggest that the dominant process by which flow modulates nanoparticle internalization is not a purely mechanical one, as included in our model, but rather an active biological phenomenon involving cellular mechanotransduction, by which the flow affects cytoskeletal organization and dynamics [9] and biochemical pathways involved in particle endocytosis [34].

## Discussion

In this section we propose simple scaling arguments to interpret our numerical results and thus to gain insight into the physics underlying the internalization process. Our goal in this section is to understand the time scales governing the kinetics of internalization, as well as the dependence of the nanoparticle internalization depth on the membrane rigidity and cytoplasmic Young’s modulus.

As shown in Fig 3, the internalization dynamics can be described as consisting of three distinct stages: development of a firm adhesion, wrapping, and stabilization at an equilibrium wrapped state. What sets the duration of these different stages? Let us first consider the time needed for completing the firm adhesion stage. The firm adhesion stage is characterized by the development of a sufficiently large bond density to deform the membrane and initiate the wrapping process. Since the initial bond density *ξ*_b_ is small compared to the total ligand and receptor densities, *ξ*_l_ and *ξ*_r_, we can characterize the short-time dynamics of bond formation by the following simplified version of Eq 10:

$$\begin{array}{c} \frac{\partial \xi_{b}}{\partial t}\approx K_{f}\xi_{l}\xi_{r}. \end{array}$$ (13)

We deduce from Eq 13 that the time needed to complete the firm adhesion stage and start deforming the membrane scales as

$$\begin{array}{c} t_{\mathrm{firm}}∼\frac{\xi_{b,\mathrm{firm}}}{K_{f}\xi_{l}\xi_{r}}, \end{array}$$ (14)

where *ξ*_b, firm_ is the bond density that needs to be attained in order to start deforming the membrane. Deformation of the membrane becomes possible when the energy stored by the bond springs reaches the energy needed to bend the membrane, i.e.:

$$\begin{array}{c} \frac{1}{2}\kappa {\left(l_{\max}-\lambda \right)}^{2}\xi_{b,\mathrm{firm}}∼\frac{1}{2}B{\left(\frac{1}{a}\right)}^{2}, \end{array}$$ (15)

from which we deduce that *ξ*_b, firm_ ∼ *B*/(*κ*(*l*_max_ − *λ*)^2^ *a* ^2^). Here, *l*_max_ is the maximum distance between the particle and the membrane at which bonds can form. Net bond formation occurs if the bond dissociation rate remains smaller than the bond formation rate, i.e., if ${\hat{K}}_{\text{r}}=K_{\text{d}}/(K_{\text{f}}\xi_{\text{l}})\leq 1$. The value of *l*_max_ is thus given by ${\hat{K}}_{\text{r}}=1$. Considering the dependence of *K*_d_ on *l* given by Eq 11, we deduce

$$\begin{array}{c} (l_{\max}-\lambda )∼\frac{k_{B}𝓣}{\kappa x_{b}}\ln(\frac{K_{f}\xi_{l}}{K_{d}^{\left(0\right)}}). \end{array}$$ (16)

For a bond spring constant *κ* = 0.01 N/m, Eq 16 yields a maximum bond deformation of the order of (*l*_max_ − *λ*) ≈ 10 nm, which is consistent with our numerical results. Combining Eqs 14 and 15, with (*l*_max_ − *λ*) given by Eq 16, we conclude that the duration of the firm adhesion stage scales as

$$\begin{array}{c} t_{\mathrm{firm}}∼\frac{B}{K_{f}\xi_{l}\xi_{r}a^{2}\kappa {\left(l_{\max}-\lambda \right)}^{2}}. \end{array}$$ (17)

For typical parameter values, Eqs 15 and 17 yield a typical bond density to start particle wrapping of *ξ*_b, firm_ ≈ 0.05*ξ*_l_, which is attained at a time *t*_firm_ ≈ 10^−3^ s, consistent with the results of the numerical model (Fig 3).

**Fig 9:**
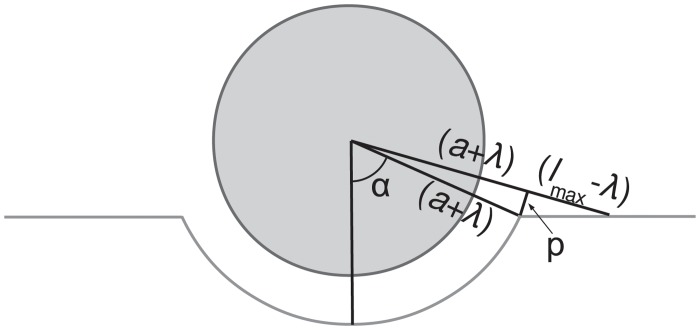
Schematics illustrating a simplified view of the geometry of early particle wrapping.

To interpret the typical duration of the wrapping phase, we propose a simplified view of the wrapping process, schematized in Fig 9. We consider a simplified geometry where the membrane is wrapped around the particle over the contact zone, adopting a spherical shape of radius *a* + *λ*, whereas outside the contact zone the membrane remains flat. Bonds follow a radial orientation. We note that this simplified view is limited to representing wrapping up to the particle’s equator, since beyond *α* = *π*/2 formation of new bonds becomes geometrically impossible (see Fig 9). At a given instant, the internalization depth *d* is related to the wrapping angle *α* by *d* = *a*(1 − cos*α*). New bonds are being formed over an annulus of width *p*, where bond length remains smaller than *l*_max_ defined above. Geometric considerations yield *p* = (*l*_max_ − *λ*)/tan*α*. Since bond formation requires a time of the order of *t*_b_ ∼ *K*_f_ *ξ*_l_, we can write the following equation of the simplified internalization dynamics:

$$\begin{array}{c} \frac{d\alpha }{dt}\approx \frac{p/(a+\lambda )}{t_{b}}=\frac{\left(l_{\max}-\lambda \right)}{\left(a+\lambda \right)t_{b}\tan\alpha }. \end{array}$$ (18)

This differential equation yields *d*/(2*a*) ∼ (cos*α*)/2 ∼ exp{[−(*l* − *λ*)(*a* + *λ*)/*t*_b_]*t*}/2, which is an exponentially asymptotic evolution equation towards an equilibrium value, here of *d*/2*a* = 0.5, and conceptually similar to the asymptotic evolution described by our computational model and shown in Fig 3. This simple reasoning suggests that the time for wrapping scales as

$$\begin{array}{c} t_{\mathrm{wrapping}}∼\frac{\left(a+\lambda \right)}{\left(l-\lambda \right)}t_{b}, \end{array}$$ (19)

which, for *a* = 50 nm, yields *t*_wrapping_ ∼ 7*t*_b_. The full computational model predicts indeed a proportionality between the two time scales, as shown in Fig 5 above, but with a coefficient of proportionality of about 25 rather than 7, corresponding to the more accurate wrapping geometry considered by the computational model.

**Fig 10:**
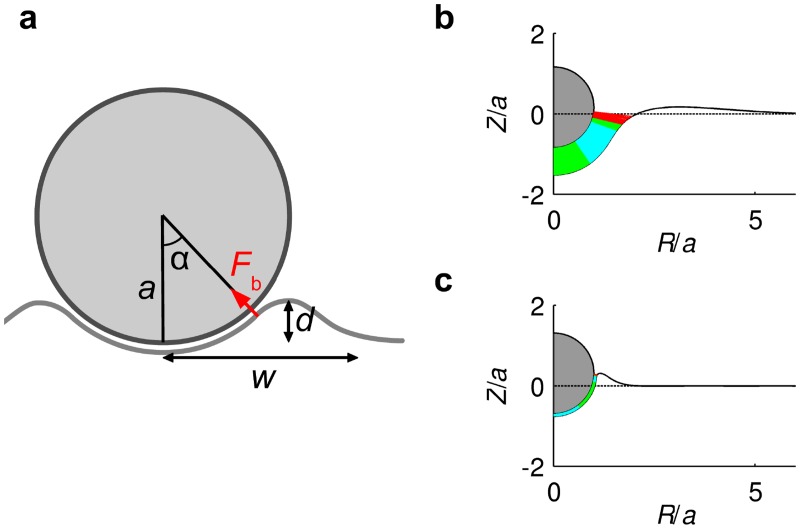
Illustration of the particle wrapping process. **a.** Schematic illustrating the geometric parameters used in the scaling arguments. **b.** Snapshot of the internalization geometry for a particle of radius *a* = 30 nm at the time when *d*/(2*a*) = 0.5. **c.** Snapshot of the internalization geometry for a particle of radius *a* = 300 nm at the time when *d*/(2*a*) = 0.5.

Next we discuss the dependence of the maximum internalization depth on the membrane and cytoplasm rigidity (*B* and *E*), presented in Fig 7 above. As shown in Fig 7a, membrane bending rigidity sets a minimum size for nanoparticle internalization, and the minimum particle size that can be internalized decreases with decreasing *B*. Here we propose a scaling argument to interpret the observed dependence. For small particles (*a* < 30 nm), the main resisting force to internalization is due to membrane bending, whereas the driving force is provided by the bonds forming at the edge of the wrapping region. Momentum balance per unit of azimuthal length yields the scaling *B*d*θ*/d*s* ∼ *F*_b_(*ξ*_l_ *p*)*w*, where *F*_b_ is the force per bond, *p* is the width of the annulus of stretched bonds at the edge of the wrapping zone (see Fig 9), and *w* is the horizontal extent of the deformed region of the membrane (see Fig 10a). The rate of change of membrane inclination scales as d*θ*/d*s* ∼ *α*/*w* ∼ (*d*/*a*)/*w*. For small particles, the two dominant terms defining the deformed shape of the membrane (see Fig 10b) are the membrane bending rigidity and the cytoplasm elasticity. Balance between these two yields a typical extent of membrane deformation of *w* ∼ (*B*/*K*)^1/4^ = (*Ba*/*E*)^1/4^. Combining these scalings leads to

$$\begin{array}{c} \frac{d}{2a}∼\sqrt{\frac{a}{BE}}. \end{array}$$ (20)

**Fig 11:**
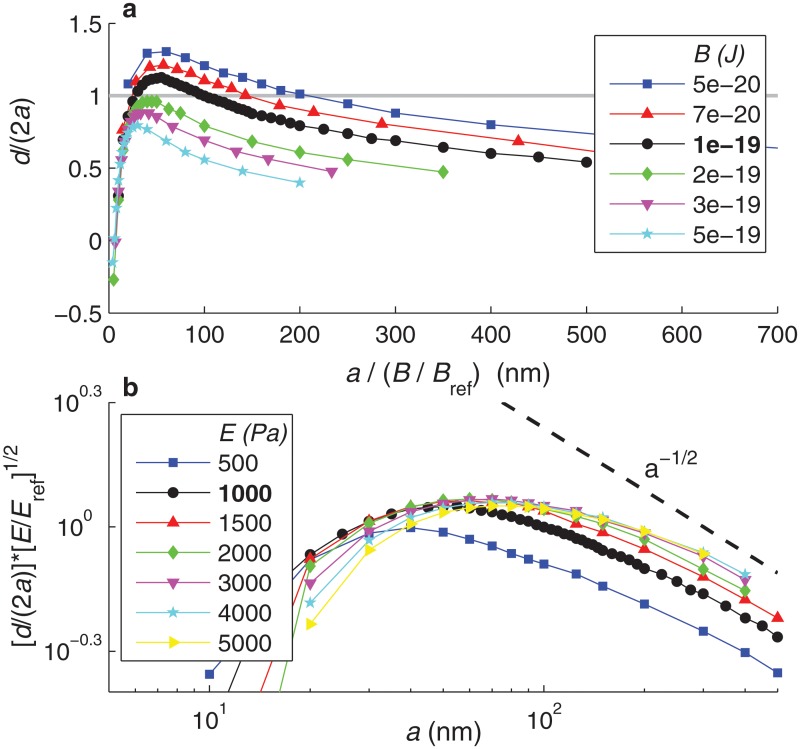
Analysis of the dependence of the maximum internalization depth on physical parameters *B*, *E*, and *a*. **a.** Dependence of the maximum internalization depth, *d*/(2*a*), on the membrane bending modulus *B*. This plot presents the results of Fig 7a, here with the horizontal axis transformed into *a*/(*B*/*B*_ref_), with *B*_ref_ ≡ 10^−19^ J. **b.** Dependence of the maximum internalization depth, *d*/(2*a*), on the cell’s Young modulus *E*. This plot presents the results of Fig 7c, here with the vertical axis scaled as (*d*/(2*a*))(*E*/*E*_ref_)^1/2^, with *E*_ref_ ≡ 1000 Pa, and the figure presented in double logarithmic scale. The dashed line indicates the slope of the power law *d*/(2*a*) ∼ *a* ^−1/2^.

Fig 11a represents the data of Fig 7a rescaled according to the dependence on *B* suggested by Eq 20. In Fig 7a the curves for different *B* collapsed at large *a*, since for large particles membrane bending rigidity becomes small compared to membrane tension and cytoplasmic elasticity, and thus its effect on internalization becomes negligible. In Fig 11a, the rescaled curves collapse at small *a*, suggesting that the scaling argument given by Eq 20 correctly captures the effect of *B* on the internalization of small particles.

As shown in Fig 7, the internalization of large particles (*a* > 100 nm) is largely controlled by the cytoplasmic Young’s modulus, *E*. We can also interpret this dependence using a scaling argument. The elastic force per unit of azimuthal length required to vertically indent the cytoplasm scales as (*Kd*) ⋅ *w*, where the first factor represents the elastic force per unit area and the second factor is the horizontal extent of the deformed region. For large particles (*a* > 100 nm), *w* ∼ *d* (see Fig 10c). Balance between the elastic force and the bond force yields the scaling *Kd* ^2^ ∼ *F*_b_ *ξ*_l_ *p*. Since *K* = *E*/*a*, we deduce a simple scaling law for the dependence of *d* on *a* and *E*, valid for large particle radius:

$$\begin{array}{c} \frac{d}{2a}∼\frac{1}{\sqrt{Ea}}. \end{array}$$ (21)

Fig 11b represents in log-log scale the data of Fig 7c rescaled according to Eq 21. This rescaling makes the different curves nearly collapse for large *a*, suggesting that Eq 21 correctly captures the effect of *E* on the internalization of large particles. Only the scaled curve corresponding to *E* = 500 Pa is far from collapsing with the others; this is attributed to the fact that, for this smaller value of *E*, the cell’s elastic rigidity is no longer the dominant resisting mechanism to nanoparticle internalization. Fig 11b also shows that, for large particles, the maximum internalization depth *d*/(2*a*) seems to decrease with $1/\sqrt{a}$, consistent with Eq 21.

## Conclusion

Our model results show the existence of an optimal radius for receptor-mediated nanoparticle internalization of the order of 50 nm. Below this optimal radius, receptor-mediated internalization becomes rapidly impaired by membrane bending rigidity. Above the optimal radius, cytoplasmic rigidity makes internalization less efficient, but the effect of increasing the radius above the optimum is more gradual, as the maximum depth to which the particle can be internalized decreases only with the inverse of the square root of the particle size. We have also shown that bond characteristics play an important role in internalization. Notably, we have identified an optimum value of the bond elastic constant, of the order of 5 nN/*μ*m, for which internalization is most efficient.

Whereas experimental studies have evidenced an effect of flow on nanoparticle internalization [9, 34], our model suggests that the mechanical effect of a shear force applied on a nanoparticle is only important in determining nanoparticle adhesion to the cell membrane. Once firm adhesion is attained, our model predicts that internalization dynamics become independent of the direct mechanical action of the shear. This result suggests that shear affects nanoparticle internalization by modulating the active dynamics of the cellular cytoskeleton, rather than by a direct mechanical effect on the nanoparticle. In contrast, our model predicts that normal compressive stresses applied to nanoparticles can significantly improve their internalization, especially of those particles larger than about 100 nm.

A limitation of the model presented here is that it disregards active forces generated by the cellular cytoskeleton during nanoparticle internalization. Indeed, experiments show that ICAM-1-mediated internalization is dependent on the formation of actin stress fibers, a phenomenon that occurs over a time scale of about 10 minutes [3]. In contrast, the internalization mechanism described here is completed over a much shorter time scale (of a minute or less). Our model thus only describes the initial step of receptor-mediated nanoparticle internalization, whereas later stages of internalization are controlled by cytoskeletal dynamics. Thus, our model can be directly validated against experiments of receptor-mediated nanoparticle uptake into artificial systems that lack a dynamic cytoskeleton, such as vesicles [35, 36] or polymersomes [37]. As our model accounts for the main internalization mechanisms with the exception of cytoskeletal dynamics, comparison between our model’s predictions and experiments of nanoparticle cell uptake can be used to isolate the contribution of cytoskeletal dynamics to the internalization process. Based on such additional experimental knowledge, cytoskeletal dynamics can be included in the model by making the cytoplasmic rheology or the bond properties depend on the internalization stage, or by adding an additional, time-dependent force acting on the membrane, to account for the active forces generated by the cytoskeleton.
